# Decoding the Dialog Between Plants and Arbuscular Mycorrhizal Fungi: A Molecular Genetic Perspective

**DOI:** 10.3390/genes16020143

**Published:** 2025-01-24

**Authors:** Vanessa Díaz, Maite Villalobos, Karem Arriaza, Karen Flores, Lucas P. Hernández-Saravia, Alexis Velásquez

**Affiliations:** 1 Laboratorio de Genómica de Ambientes Extremos, Facultad de Recursos Naturales Renovables, Universidad Arturo Prat, Campus Huayquique, Iquique 1100000, Chile; vadiazm@estudiantesunap.cl (V.D.); mvillalobosp@estudiantesunap.cl (M.V.); 2 Núcleo de Investigación Aplicada e Innovación en Ciencias Biológicas, Facultad de Recursos Naturales Renovables, Universidad Arturo Prat, Campus Huayquique, Iquique 1100000, Chile; 3 Centro de Investigación en Medicina de Altura, Universidad Arturo Prat, Iquique 1100000, Chile; karriaza@unap.cl (K.A.); kfloresu4859@universidadarturoprat230.onmicrosoft.com (K.F.); 4 Laboratorio de Laboratorio de Bionanomateriales, Facultad de Recursos Naturales Renovables, Universidad Arturo Prat, Av. Arturo Prat s/n, Campus Huayquique, Iquique 1100000, Chile

**Keywords:** small RNA, GRAS transcription factor, arbuscular mycorrhizal symbiosis, strigolactones, plant–mycorrhizal interaction

## Abstract

Arbuscular mycorrhizal (AM) symbiosis, a mutually beneficial interaction between plant roots and AM fungi, plays a key role in plant growth, nutrient acquisition, and stress tolerance, which make it a major focus for sustainable agricultural strategies. This intricate association involves extensive transcriptional reprogramming in host plant cells during the formation of arbuscules, which are specialized fungal structures for nutrient exchange. The symbiosis is initiated by molecular signaling pathways triggered by fungal chitooligosaccharides and strigolactones released by plant roots, which act as chemoattractants and signaling molecules to promote fungal spore germination, colonization, and arbuscule development. Calcium spiking, mediated by LysM domain receptor kinases, serves as a critical second messenger in coordinating fungal infection and intracellular accommodation. GRAS transcription factors are key components that regulate the transcriptional networks necessary for arbuscule development and maintenance, while small RNAs (sRNAs) from both plant and fungi, contribute to modifications in gene expression, including potential bidirectional sRNA exchange to modulate symbiosis. Understanding the molecular mechanisms related to AM symbiosis may provide valuable insights for implementation of strategies related to enhancing plant productivity and resilience.

## 1. Introduction

The intricate ecological interplay between plants and microorganisms, specifically rhizospheric microorganisms, has a substantial impact on plant physiology and agricultural outcomes. These microorganisms can boost plant performance by enhancing nutrient acquisition, stress tolerance, and overall growth, thereby contributing to more sustainable and productive agricultural systems [1,2].

**Figure 1:**
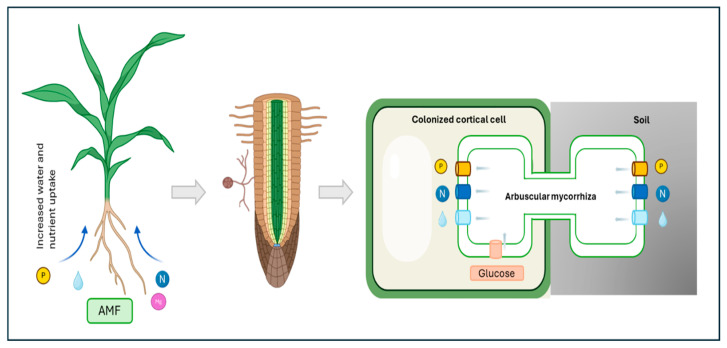
Nutrient exchange between plants and arbuscular mycorrhizal fungi. The mycorrhizal fungus provides minerals and water to plants, which in exchange provide a fixed carbon source to the fungus.

Arbuscular mycorrhizal fungi (AMF) are obligate biotrophic organisms that belong to the *Glomeromycota* phylum. These fungi form one of the most widespread symbiotic associations with plant roots [3]. This ancient symbiosis is characterized by a complex molecular dialogue between the plant and the fungus, leading to the formation of specialized structures within plant roots. These structures, known as arbuscules, serve as the primary site for nutrient exchange [4]. AMF enhance plant nutrient uptake, particularly phosphorus and nitrogen, and improve plant tolerance to various biotic and abiotic stresses. In return, plants provide carbon-based compounds to the fungi (Figure 1).

The establishment of the AM symbiosis requires a complex chemical crosstalk between the plant host and its fungal partner. During the pre-symbiotic phase, plant roots release signaling molecules called strigolactones into the rhizosphere, which induce fungal spore germination and hyphal branching. In response, AM fungi release chitooligosaccharides (COs) and lipochitooligosaccharides (LCOs), signaling molecules that are recognized at the plant plasma membrane [5]. This recognition initiates a cascade of signaling events that promote fungal colonization of the root and induce extensive transcriptional reprogramming in the host plant. A central pathway involved in this signaling process is the common symbiosis signaling pathway, also known as the sym pathway, which is required for both mycorrhization and nodulation in legume plants [6]. Nodulation is a symbiotic process that occurs primarily in legume plants such as peas, beans, clover, and alfalfa. It involves a mutually beneficial relationship between the plant and nitrogen-fixing bacteria, mainly *Rhizobium* species. *Medicago truncatula* has been extensively studied as a model legume for investigating the molecular genetics of nitrogen-fixing root–nodule symbiosis [6]. One of the key components of the sym pathway is the induction of perinuclear calcium oscillations in the host cell. These calcium signals activate a calcium- and calmodulin-dependent serine/threonine protein kinase known as CCaMK, which, in turn, triggers the transcriptional changes necessary for the establishment of the symbiotic interface [7]. This interface, called the peri-arbuscular membrane, envelops the arbuscule and provides a site for bidirectional nutrient and signal exchange. The formation and maintenance of the peri-arbuscular membrane require significant structural and functional modifications in the host root cells, indicating a high degree of coordination between plant and fungal gene expression. The composition of the peri-arbuscular membrane is distinct from the typical plasma membrane, containing specific proteins and lipids that facilitate the efficient transfer of nutrients, such as phosphate transporters and ammonium channels. These molecular components ensure that the symbiotic relationship remains beneficial and controlled, preventing any overexploitation by the fungal partner [8,9].

**Figure 2:**
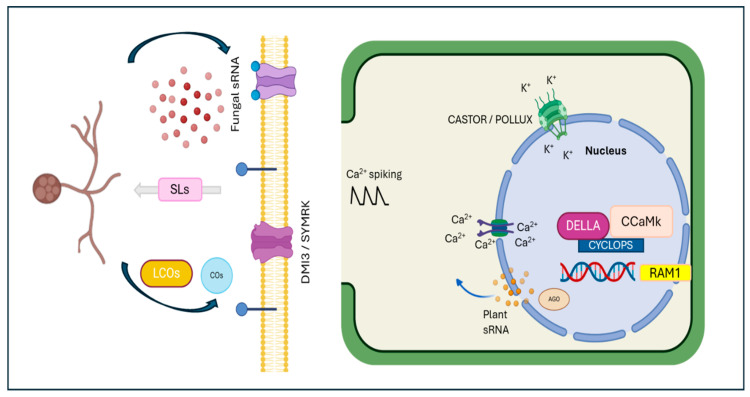
Establishment of arbuscular mycorrhizal interaction. Plant roots release strigolactones, which induce arbuscular mycorrhizal spore germination and hyphal branching. The mycorrhizal fungi produce Myc factors, which are recognized by the DMI3/SYMRK receptor complex. This stimulus activates perinuclear calcium spiking, involving the CASTOR and POLLUX channels. Required for arbuscular mycorrhization 1 (RAM1) is essential during the early colonization stage, while the DELLA/CYCLOPS/CCaMK complex is necessary for arbuscule development. Additionally, both plant and fungal sRNAs are involved in mycorrhizal symbiosis, targeting various genes and regulating their expression—a crucial process for maintaining symbiosis.

AM colonization not only affects local root physiology but also induces systemic changes in the plant, impacting nutrition, development, and responses to both biotic and abiotic stresses. These systemic effects imply a root-to-shoot signaling axis that influences multiple aspects of plant biology. Metabolic changes in shoots are often observed in plants undergoing AM colonization, suggesting a coordinated response across the entire plant [10]. Additionally, systemic changes influence the plant’s ability to cope with environmental stresses, indicating that AM symbiosis has extensive benefits beyond the root system. For example, AM colonization can improve drought tolerance by enhancing water uptake and increasing the production of stress-related proteins and metabolites. It also aids in the uptake of less-mobile nutrients, such as zinc and copper, which are crucial for various plant metabolic processes [11,12]. Recent research has also highlighted the role of small RNAs (sRNAs) in regulating the AM symbiosis. These molecules are involved in regulating gene expression in both the plant and fungal partners. Some sRNAs have been found to act as mobile signals that can move between the plant and the fungus, influencing the symbiotic relationship at both local and systemic levels (Figure 2). This cross-kingdom RNA interference is a promising area of research that could unlock new strategies for optimizing AM symbiosis for agricultural benefit. The aim of this review was to delve deeper into the key molecular processes involved in the AMF–plant interaction. By understanding the molecular mechanisms underlying this sophisticated symbiotic relationship, different strategies to enhance plant growth and productivity may be developed, especially in challenging environmental conditions.

## 2. Signaling Molecules in the Establishment of Symbiotic Associations

Chitin, a polymer of N-acetylglucosamine, and its derivative chitosan are essential components of fungal cell walls and central to plant–fungal interactions. In rhizobium–legume associations, flavonoids, isoflavonoids, and betaines produced by legumes induce the expression of nod genes in rhizobia, leading to the synthesis of LCOs, which play a major role in symbiotic association [13,14]. Plants also recognize fragments of chitin, called COs, as microbe-associated molecular patterns, triggering immune responses such as gene expression, activation of mitogen-activated protein kinases, production of reactive oxygen species (ROS), and calcium influx. Eight-residue COs are particularly effective in activating these immune responses, and their detection involves receptor-like kinases containing LysM domains [15]. For instance, in *M. truncatula*, the MtLYK9/MtCERK1 and MtLYR4 receptor complex binds eight-residue COs, initiating immune signaling [16,17]. Similarly, other plants like rice and *Arabidopsis thaliana* utilize analogous receptor complexes to recognize COs and activate defense mechanisms [18]. Nevertheless, COs are also produced by beneficial symbionts, such as AMF, compelling a balance between immune responses and symbiotic signaling [18]. The original paradigm proposed that long-chain COs stimulate immunity, whereas short COs initiate symbiotic pathways. However, recent studies have revealed a more complex interaction, demonstrating that long COs can activate both immune and symbiotic responses. In *M. truncatula*, eight-residue COs can induce calcium spiking and upregulate symbiosis-related genes, mediated by the same receptor complex that detects immune signals, highlighting the important role of COs recognition in host discrimination [17].

AMF synthesize a mixture of COs and LCOs, both of which contribute to symbiotic signaling. Sulfated LCOs are particularly effective, surpassing non-sulfated LCOs and COs in triggering calcium spiking and promoting fungal colonization in various plant species. For example, in *M. truncatula*, sulfated LCOs stimulate calcium spiking in root hairs and atrichoblasts of lateral roots, which are the main sites for fungal colonization [19]. In non-leguminous plants like French marigold and organ culture carrot, treatments combining sulfated and non-sulfated LCOs enhance root colonization and calcium spiking, underscoring the importance of LCOs in establishing AM symbiosis [19,20]. Moreover, AM fungal LCOs suppress defense responses to COs, facilitating symbiotic interactions. Interestingly, LCOs from non-mycorrhizal fungi can also induce early symbiotic responses, such as root hair branching in *M. truncatula* and *Vicia sativa*, suggesting that LCO synthesis is a conserved fungal trait [14,19]. Mass spectrometry analyses of fungal exudates reveal that fungal LCOs share structural similarities with those of AMF, indicating a conserved and versatile role in plant–fungal communication. These findings highlight the dual nature of COs and LCOs in modulating plant immunity and symbiosis, emphasizing the intricacies of their molecular interplay.

The interaction between COs and LCOs illustrates a sophisticated mechanism by which plants discern between pathogenic and symbiotic fungi, fine-tuning their responses to optimize both defense and symbiotic efficiency. For instance, while eight-residue COs trigger immune signaling, the presence of LCOs can suppress these defenses, allowing AMF to establish colonization and promote nutrient exchange [17]. This delicate balance between immunity and symbiosis is mediated by calcium spiking, a pivotal second messenger in both pathways, and receptor complexes capable of integrating diverse molecular signals. Understanding these dynamics provides valuable insights into the evolutionary and functional significance of chitin derivatives in plant–fungal interactions [14]. The ability of AMF to modulate plant responses through COs and LCOs underscores their critical role in promoting plant health and productivity. These molecules not only enhance nutrient acquisition by facilitating AM symbiosis but also influence root development, such as lateral root formation, further supporting fungal colonization [21].

While fungi produce LCOs and COs to establish symbiotic associations, plants produce key molecules called strigolactones (SLs). SLs are a group of carotenoid-derived phytohormones exuded by plant roots. These molecules serve dual roles as developmental regulators within plants and as signaling compounds that mediate interactions with other organisms in the soil. Initially discovered as germination stimulants for parasitic weeds, such as those in the genus Striga [22], SLs have been then recognized as key players in plant architecture regulation and facilitators of AMF development [23]. AMF rely on SLs to induce hyphal growth and branching, enabling them to establish symbiotic relationships with host plants. This interaction is particularly well-documented in members of the *Gigasporaceae* and *Glomeraceae* families, where SLs stimulate processes such as hyphal proliferation, mitochondrial enlargement, and increased ATP content [24,25,26]. Interestingly, other root-exuded compounds, like hydroxy fatty acids, can also induce hyphal branching, suggesting that SLs are part of a broader signaling network [27]. The structural composition of SLs is central to their bioactivity. These molecules consist of a tricyclic lactone connected to a methylbutenolide via an enol ether bond, with both components being essential for their function. Modifications to the A and B rings, such as the presence of hydroxyl, keto, or acetyl groups, significantly influence their activity [28]. Plant genetic studies have identified key components in SL biosynthesis and secretion. For instance, the G-subfamily ABC transporter PDR1 in *Petunia* mediates the exudation of orobanchol, which is a prominent SL. Mutations in PDR1 result in reduced SL release, impairing AMF colonization due to lower hyphopodium initiation and intraradical fungal spread [29]. The localized expression of PDR1 in hypodermal passage cells, which serve as entry points for AMF into the root cortex, underscores the targeted nature of SL signaling [30].

SL biosynthesis is tightly regulated and responsive to environmental stimuli, particularly phosphate availability. Under phosphate starvation, plants boost SL production to enhance AMF colonization, as the symbiosis aids in phosphate acquisition [31]. This process is coordinated by GRAS-type transcription factors, notably NSP1 and NSP2, which regulate the expression of DWARF27 (D27), encoding a β-carotene isomerase critical for the initial steps of SL biosynthesis [32,33]. Mutants deficient in NSP1 or NSP2 exhibit disrupted SL biosynthesis and exudation, leading to compromised AMF colonization. Notably, in *Medicago*, NSP2 is regulated by microRNA (miRNA) miR171h, which restricts colonization to specific root zones by locally reducing SL biosynthesis. Overexpressing a miR171h-resistant version of NSP2 enables colonization of typically uncolonized root elongation zones, revealing a fine-tuned mechanism of spatial regulation in SL-mediated interactions [34]. The perception of SLs involves the α/β-hydrolase receptor D14/DAD2/HTD2, which interacts with the F-box protein D3/MAX2/RMS4. Binding of SLs to D14 triggers a conformational change, facilitating its interaction with D3 and subsequent activation of an E3 ligase, which targets specific proteins for degradation to enable downstream signaling [35]. The F-box protein D3 is essential for AMF colonization, as evidenced in rice and pea mutants lacking this protein. These mutants fail to support AMF accommodation and expression of symbiosis-related genes, despite showing enhanced hyphal branching due to elevated SL exudation [36,37]. Intriguingly, SL signaling is not always required for AMF colonization, as demonstrated in rice D14 mutants, which exhibit higher colonization levels likely due to increased SL release. This suggests that D3 may participate in additional pathways that promote symbiosis, independent of SL signaling [37,38].

## 3. Calcium Spiking in Response to Symbiosis

Calcium spiking is a critical component of plant cellular signaling, which involves dynamic and repetitive increases in calcium concentration within the nucleus and perinuclear cytoplasm. These calcium oscillations originate from calcium stores located near the nuclear envelope, likely involving the endoplasmic reticulum [7]. To facilitate calcium spikes, it is hypothesized that a core molecular machinery is necessary, comprising calcium channels for release, counter-ion channels such as potassium or anion channels to balance charges during release, and calcium ATPases that pump calcium back into the store for replenishment [39]. Among these key components, the CASTOR and POLLUX channels, also known as MtDMI1, has been identified to be implicated as counter-ion channels. Similarly, the SERCA-type ATPase MCA8 is proposed as a nuclear-localized calcium pump responsible for restoring calcium levels in these stores [40]. Additional insights reveal that mutations in nucleoporin genes—NUP85, NUP133, and NENA—disrupt calcium spiking. These genes belong to the NUP107 subcomplex, a group of nuclear pore proteins potentially involved in trafficking essential components of the spiking machinery, such as channels and pumps, to their functional sites in the inner nuclear envelope [39]. However, the precise mechanisms regulating this transport remain unclear.

Calcium signaling plays a pivotal role in symbiotic interactions between plants and microorganisms, such as rhizobia and AMF. Upon recognizing these symbiotic partners, plants initiate calcium oscillations in the nucleus that is one of the earliest detectable responses [27]. For rhizobia, this process begins with the perception of Nod factors by the plant, triggering calcium spikes within minutes [41]. The involvement of plasma membrane-bound receptor complexes, as well as additional components associated with the nuclear envelope, underscores the complexity of this signaling pathway. Similarly, calcium spiking is observed in plant roots responding to AMF, even before direct contact with fungal hyphae [40]. This suggests that diffusible fungal signals, such as Myc factors, play a role in activating the calcium response. Among these signals are LCOs and COs which are considered as key molecules. Fungal-derived sulfated and non-sulfated LCOs, along with short-chain COs, have been shown to induce calcium oscillations in plant root epidermal cells. Interestingly, the calcium responses elicited by AMF and rhizobia exhibit notable similarities, with subtle differences in spiking frequency and duration [42]. While fungal and short-chain CO-induced oscillations tend to have more variability and longer durations compared to those induced by Nod factors or LCOs, their overall structural profiles are largely similar. These findings suggest that the specific composition and relative concentrations of these signaling molecules determine the calcium response’s characteristics. Moreover, calcium spiking patterns evolve during symbiotic colonization. For instance, low-frequency oscillations are observed in the outer cortical cells before infection, whereas higher-frequency spiking occurs during the entry of symbionts into host cells [43]. This progression implies that calcium signaling is tightly regulated and adjusts to the stages of symbiotic development, coordinating cellular reprogramming in the host plant to accommodate the symbiotic process.

These responses underscore the importance of calcium signaling in plant ability to distinguish between different symbiotic microorganisms at early interaction stages. While it was initially hypothesized that rhizobia and AMF might induce distinct calcium spiking signatures, subsequent studies revealed that these responses are remarkably similar, particularly when induced by LCOs [39,44]. However, the variability and longer duration of fungal-induced calcium spiking suggest a degree of signaling specificity. Additionally, short-chain COs and LCOs exhibit cell-type-specific activation patterns, with responses differing between trichoblasts and atrichoblasts depending on their combinations and concentrations. For instance, in rice, calcium spiking in trichoblasts is only observed when both COs and LCOs are present, emphasizing the combinatorial character of these signals [42,45].

## 4. Role of GRAS-Domain Proteins in Arbuscular Mycorrhizae

GRAS proteins form a diverse family of plant-specific transcription factors (TFs) with essential roles in a wide range of developmental processes, signaling pathways, and environmental responses. These proteins typically consist of 360 to 850 amino acids and are characterized by a conserved GRAS domain at the C-terminal region, which includes motifs such as VHIID, SAW, and PFYRE, as well as two leucine heptad repeats (LHR). This conserved structure, first detailed by [46], contrasts with the N-terminal region, which is variable and often intrinsically disordered, allowing GRAS proteins to interact with a wide variety of other proteins [47]. One notable feature of some GRAS proteins is the DELLA motif in their N-terminal region, which modulates interactions with other transcription factors, as demonstrated by [48]. These structural characteristics contribute to the multifaceted roles of GRAS proteins in plant biology. The acronym GRAS derives from the first identified members of this family: gibberellin acid insensitive (GAI), repressor of GA1 (RGA), and scarecrow-like (SCL) proteins [46]. These proteins serve as key regulators in critical processes such as gibberellin signaling, root and shoot development, light and stress responses, and symbiotic interactions with microorganisms [49,50,51]. The involvement of GRAS proteins in gibberellin acid signaling, for instance, is exemplified by DELLA proteins, which act as repressors in the GA pathway by interacting with other proteins, including those from the indeterminate domain (IDD) family [52].

Genome-wide analyses have revealed 29 orthologous groups across several species [53]. These groups have been classified into 17 subfamilies, with 5 newly established ones, including RAD1 and RAM1, which are implicated in mycorrhizal signaling [54,55]. Interestingly, these subfamilies exhibit taxonomic specificity, as demonstrated by the absence of some GRAS subfamilies in *Brassicales*. Such findings underscore the evolutionary diversification of GRAS proteins and their specialized roles in distinct plant lineages. The roles of GRAS proteins extend beyond individual pathways; they often function within complex regulatory networks. For example, the interplay between SCARECROW (SCR) and SHORT-ROOT (SHR) transcription factors in radial root organization has been well-studied [56]. Additionally, SCL3 mediates gibberellin-promoted cell elongation in roots, acting as a bridge between the SCR/SHR and GA/DELLA pathways [57,58]. These interactions illustrate the ability of GRAS proteins to integrate hormonal and developmental signals, ensuring coordinated growth and differentiation. One of the most intriguing aspects of GRAS protein functionality is their role in symbiotic processes, particularly in mycorrhizal associations. GRAS proteins such as RAM1 and RAD1 have been identified as central regulators of arbuscular mycorrhiza [59,60]. These transcription factors are essential for the establishment and maintenance of AM symbiosis, facilitating the accommodation of fungal structures within host root cells. RAM1, for instance, activates the expression of genes involved in lipid and carbohydrate metabolism, as well as those encoding membrane proteins critical for arbuscule function [61,62]. Furthermore, RAM1 interacts with other GRAS-domain proteins, including RAD1, to form regulatory networks that modulate arbuscule development [63].

DELLA proteins also play a dual role in AM symbiosis, promoting arbuscule formation while contributing to arbuscule degeneration through specific regulatory complexes. For instance, DELLA forms a complex with CYCLOPS and other proteins to activate RAM1 transcription, thereby initiating arbuscule formation [60]. Conversely, DELLA interacts with NSP1 and MYB1 to regulate arbuscule degeneration, highlighting its dynamic involvement in different stages of the symbiotic lifecycle [64]. These contrasting roles emphasize the complexity of GRAS-mediated regulation in symbiosis. In legumes, GRAS proteins such as NSP1 and NSP2 are integral to both nodulation and mycorrhization. These transcription factors form a complex that activates early nodulation genes and strigolactone biosynthesis, a process critical for AM fungal colonization [20,23]. Mutants lacking NSP1 or NSP2 exhibit reduced mycorrhizal colonization, demonstrating the essential role of these proteins in symbiotic signaling [65]. Additionally, NSP2 interacts with RAM1 to regulate mycorrhizal gene expression, providing further evidence of the interconnected roles of GRAS proteins in symbiotic pathways [66]. The regulatory versatility of GRAS proteins is further exemplified by their ability to form heterodimers, which are often essential for their functionality [66]. Genome analysis of the GRAS gene family highlights the combinatorial interactions between GRAS proteins and other transcriptional regulators, which enable them to participate in diverse processes without directly binding DNA [49]. This capacity for protein–protein interaction allows GRAS proteins to act as transcriptional regulators, forming complexes that modulate gene expression in a context-dependent manner. In addition to their established roles in GA signaling and symbiosis, GRAS proteins are implicated in stress responses and light signaling. For instance, they contribute to the regulation of root architecture under abiotic stress conditions, adapting plant growth to environmental challenges [49]. The involvement of GRAS proteins in light signaling further demonstrates their versatility, as they integrate external signals to optimize plant development and resource allocation. Nevertheless, the molecular mechanisms underlying their interactions with other proteins and their regulation by post-transcriptional and post-translational modifications are not fully understood. Recent studies have identified miRNA that target GRAS genes, such as miR171h, which regulates NSP2 expression during mycorrhization [34]. Such findings suggest additional layers of regulatory complexity that warrant further investigation.

## 5. Small RNAs in Mycorrhizal Symbiosis

RNA interference (RNAi) is a conserved regulatory mechanism across eukaryotes, playing a crucial role in development and stress responses [67]. This process is mediated by sRNAs, which guide gene silencing either transcriptionally (TGS) or post-transcriptionally (PTGS), depending on sequence complementarity. Plant sRNAs, typically 20–28 nucleotides long, are classified into miRNAs and short interfering RNAs (siRNAs) based on their biogenesis [68]. miRNAs are derived from single-stranded RNAs forming stable hairpin structures and are processed by the DICER-like enzyme DCL1. These miRNAs predominantly facilitate PTGS by cleaving target mRNAs or repressing their translation [69]. SiRNAs, in contrast, originate from double-stranded RNAs (dsRNAs) that may be endogenous or exogenous, processed by RNA-dependent RNA polymerases (RdRps). SiRNAs contribute to PTGS via 21–22 nucleotide siRNAs and TGS through 24 nucleotide heterochromatic siRNAs (hc-siRNAs), which guide RNA-directed DNA methylation [70]. Once synthesized, sRNA duplexes are stabilized through methylation by the HUA ENHANCER1 (HEN1) enzyme and incorporated into the RNA-induced silencing complex (RISC), where ARGONAUTE (AGO) proteins execute RNAi functions. AGO proteins, highly conserved across eukaryotes, vary in number and function among plant species. In *A. thaliana*, AGO proteins are grouped into three clades (AGO1/5/10, AGO2/3/7, and AGO4/6/8/9) and demonstrate functional specialization based on the sRNA length, 5′ nucleotide, and duplex structure [71,72]. Interestingly, sRNAs and AGO proteins often function beyond their originating cells, utilizing extracellular vesicles (EVs) or apoplastic spaces for transport. Unique RNAi components in certain plants, such as the monocot-specific DCL5 involved in temperature-dependent fertility or the unusually long sRNAs in *Chlamydomonas reinhardtii* AGO1, underscore evolutionary adaptations [71,73].

Plant sRNAs play a pivotal role in plant–biotic interactions, particularly in symbiosis and pathogen defense. They regulate endogenous genes, safeguard genome integrity from transposable elements, and defend against viral infections. Viral dsRNAs are processed into viral sRNAs by host RNAi machinery, which targets viral genomes to curtail infections or, in some cases, modulates host transcripts for viral persistence [74]. Beyond viruses, plant sRNAs respond to bacterial and fungal elicitors, such as flagellin and chitin, activating antimicrobial defenses [75,76]. Plant sRNAs can even translocate into pathogens, impacting their virulence; for example, miR159 and miR166 from cotton target *Verticillium dahliae* virulence genes [77]. This trans-kingdom RNAi phenomenon has inspired further exploration of plant-derived sRNAs for antimicrobial applications [78,79].

AMF colonization significantly reprograms sRNA expression in plants, a phenomenon conserved across species [63,80,81]. In *M. truncatula*, miRNAs affected by AM colonization target transcription factors that regulate AM development and immune suppression [82]. Similarly, in *Nicotiana attenuata*, AGO7 regulates AM-induced sRNAs, influencing colonization through phytohormone signaling and nutrient metabolism pathways [83]. Loss of AGO7 enhances mycorrhizal colonization, paralleling observations in *M. truncatula* mutants where nodulation is similarly increased [84], suggesting shared mechanisms between symbiotic interactions. Despite progress, mechanistic insights into miRNA roles in AM symbiosis are limited. For example, miR399, a key player in phosphate starvation signaling, modulates AM colonization by repressing PHO2, although its overexpression cannot overcome high-phosphate suppression of AM symbiosis [85,86]. Evidence points to a broader regulatory network involving phosphate starvation response components such as PHR in orchestrating AM colonization [87]. Unlike bacterial and fungal pathogen responses, where miRNA regulation suppresses interactions, auxin signaling positively regulates AM symbiosis. Downregulation of miR393, for instance, enhances auxin signaling essential for arbuscule formation [88], whereas miR396b overexpression reduces mycorrhizal colonization by targeting transcription factors in the GRF and bHLH families [89].

Certain miRNAs exhibit spatially localized expression patterns that restrict fungal colonization. In *M. truncatula*, miR171 isoforms like mtr-miR171h target NSP2, a GRAS transcription factor necessary for AM gene expression, limiting fungal colonization to specific root zones [90]. Similarly, miR171b in AM-compatible plants protects positive regulators like LOM1 from silencing, ensuring AM compatibility [91]. *Populus trichocarpa* also shows extensive miRNA reprogramming during AM colonization, with conserved expression patterns across mycorrhizal types. Systemic responses, such as miRNA changes in leaves, suggest a potential priming effect against foliar pathogens [91]. Future research integrating diverse RNA types, such as tiny and circular RNAs, alongside detailed studies of DCL and AGO family roles, could further elucidate the complex regulatory networks underlying AM symbiosis [92,93].

Nearly all fungal lineages, including arbuscular mycorrhizal (AM) fungi, contain key components of RNA interference (RNAi) machinery, such as Dicer-like (DCL) proteins, Argonaute (AGO) proteins, and RNA-dependent RNA polymerases (RdRps). These components are often encoded by multiple genes per genome [94]. However, AMF exhibit unique adaptations in their RNAi-related gene repertoire, setting them apart from other fungi [95,96,97]. A particularly striking example is the extensive expansion of AGO genes in *Rhizophagus irregularis*, which possesses 26 full-length AGO genes and an additional 14 partial AGO sequences containing the PIWI domain but missing other domains. Furthermore, the RdRp family in *R. irregularis* includes 21 genes, some of which encode peptides with unusually short and unique RdRp domains [95]. These expansions coexist with the presence of one to two DCL genes and a unique prokaryotic-type class I ribonuclease III enzyme, likely acquired via horizontal gene transfer from cyanobacteria, which may assist in small RNA (sRNA) production [98]. Additionally, AMF retain the sRNA methyltransferase HEN1, which stabilizes sRNAs, a gene lost in several other fungal lineages [99]. Expression studies through transcriptomics and proteomics confirm that RNAi-related genes in AMF are actively expressed and regulated throughout their life cycle [95,96,99]. Sequencing of sRNAs has revealed that AMF, including *R. irregularis* and *Gigaspora margarita*, produce sRNAs with characteristic lengths of 24–26 nucleotides, consistent with the activity of a single DCL enzyme. These sRNAs exhibit a 5′ uracil bias, and some are 2′-O-methylated at the 3′ end—a modification mediated by HEN1 homologs [99]. Genomic analyses have identified two primary types of sRNA-generating loci: those associated with transposable elements (TEs), which produce 22–26 nt sRNAs, and others linked to protein-coding genes that generate sRNAs of variable lengths [95,96]. Moreover, a noncanonical, DCL-independent pathway, previously described in the non-mycorrhizal fungus *Mucor circinelloides*, might also function in AMF. This pathway relies on a prokaryotic-derived RNase III enzyme and could contribute to sRNA production in AMF [100,101].

TEs play a significant role in the genome dynamics of AMF, serving both as sources and targets of RNAi-mediated silencing. In *R. irregularis*, nearly half the genome comprises repetitive elements, with TEs contributing to 49% of the total sRNA pool. The remaining sRNAs originate from either unannotated genomic regions (41%) or protein-coding genes (10%), many of which are located near TEs, suggesting potential regulatory interactions [99]. The expansion of AGO genes in *R. irregularis* is thought to have co-evolved with TEs, reflecting their intertwined evolutionary dynamics. Besides RNAi, AMF also use DNA methylation to regulate TEs, with 5-methylcytosine (5mC) at CG sites serving as a key epigenetic mark. In addition, AMF exhibit a unique epigenetic feature: N6-methyladenine (6mA) at ApT motifs, which is primarily associated with increased transcription and genes related to symbiosis [102].

At the chromosomal level, *R. irregularis* nuclei are organized into distinct euchromatic and heterochromatic compartments. Gene-rich euchromatic regions house core functions, while heterochromatic regions contain repetitive sequences and genes encoding secreted proteins that are highly expressed during symbiosis [103,104]. This chromatin organization underscores dynamic interactions between fungal genomes and host plants, with evidence that host plants can induce changes in fungal chromatin and DNA methylation. Conversely, AMF can influence host epigenetics, as demonstrated by the effector protein RiNEL1 from *R. irregularis*, which interacts with plant histone H2B to suppress defense gene expression and enhance symbiosis [105]. AMF also seem to employ RNAi as a defense mechanism against viruses, as evidenced by the presence of sRNAs mapping to viral genomes within these fungi. However, the roles of mycoviruses in AM fungal biology remain poorly understood [95]. Beyond self-defense, AMF may engage in cross-kingdom RNAi, where sRNAs are exchanged between species to regulate gene expression. In pathogenic systems, fungal sRNAs derived from TEs can suppress host defense genes to facilitate infection [106]. Mutualistic interactions may also involve cross-kingdom RNAi, as seen in ectomycorrhizal fungi, where fungal sRNAs target plant genes to sustain the symbiosis [107]. Although direct evidence for cross-kingdom RNAi in AM symbiosis is limited, related studies demonstrate its feasibility. For instance, host-induced gene silencing (HIGS) has been used to manipulate fungal gene expression in AMF. Silencing the *R. irregularis* monosaccharide transporter gene *MST2* in *M. truncatula* roots reduced fungal colonization [108]. Similar methods have validated functions of other fungal genes related to phosphate transport, effectors, and MAPK cascades [109,110]. A computational and experimental study has also identified fungal sRNAs that target host genes, such as an *R. irregularis* sRNA that modulates *M. truncatula* WRKY transcription factors to regulate colonization [111]. Fungal sRNAs often target host immune genes like MAPK cascade components and WRKY transcription factors, while in mutualistic AM symbioses, fungal sRNAs may modulate host defense responses and membrane remodeling for arbuscule development. Recent studies suggest additional complexities, including the potential for mRNA transfer between interacting organisms, the involvement of host AGO4 proteins in driving transcriptional gene silencing via methylation, and the transport of sRNAs within extracellular vesicles, broadening the scope of inter-organismal communication beyond canonical cross-kingdom RNAi [111].

## 6. Conclusions and Future Prospects

The establishment and maintenance of AMF symbiosis relies on a complex network of molecular interactions coordinated by signaling molecules, calcium spiking, GRAS transcription factors, and small RNAs (sRNAs). Communication between plants and AM fungi hinges on the exchange of signaling molecules, including chitin derivatives (COs and LCOs) and SLs, which enable plants to discriminate between beneficial and pathogenic fungi. These molecules trigger calcium spiking, a crucial early signaling event characterized by dynamic calcium oscillations regulated by core machinery involving calcium channels, counter-ion channels, and ATPases. Notably, GRAS-domain proteins RAM1, RAD1, NSP1, and NSP2 play central roles in AM development and function, influencing fungal accommodation, arbuscule formation, and coordinating symbiotic signaling with other pathways, such as hormone signaling. Acting as key gene expression regulators, sRNAs mediate plant responses to AM colonization by influencing nutrient transport, hormone signaling, and defense pathways. Concurrently, AM fungi possess adapted RNAi machinery potentially involved in modulating host silencing and defending against mycoviruses. Future research should prioritize several key areas, including a deeper understanding of the crosstalk between CO/LCO and SL signaling pathways, as well as the role of other root exudates. Further investigation of the downstream signaling cascades triggered by these molecules, including calcium signaling and transcriptional regulation, is essential, particularly regarding the mechanisms regulating the transport of calcium spiking machinery components and the functional consequences of differing spiking dynamics between rhizobial and AM fungal interactions. Regarding GRAS-domain proteins, future research should address post-transcriptional and post-translational modifications, as well as the roles of different GRAS subfamilies in various plant species. Finally, functional characterization of diverse sRNA populations and RNAi components in both plants and AM fungi is crucial, with emphasis on the mechanisms of sRNA exchange (cross-kingdom RNAi) via extracellular vesicles, validation of in silico sRNA–mRNA interactions, and exploration of mycovirus-mediated modulation. These investigations will provide a more comprehensive understanding of AM symbiosis and provide a framework for developing strategies to enhance plant–AMF interactions for sustainable agriculture in a context of climate change.

## Data Availability

No new data were created or analyzed in this study. Data sharing is not applicable to this article.
